# Housing Satisfaction of Psychiatric Patients in Different Forms of Housing—A Cross-Sectional Study in Berlin, Germany

**DOI:** 10.3389/fpsyt.2021.652565

**Published:** 2021-06-08

**Authors:** Stefan Gutwinski, Ella Westerbarkey, Meryam Schouler-Ocak, James K. Moran, Stefanie Schreiter

**Affiliations:** ^1^Department of Psychiatry and Psychotherapy, Psychiatrische Universitätsklinik der Charité im St. Hedwig-Krankenhaus, Berlin, Germany; ^2^Charité—Universitätsmedizin Berlin, Corporate Member of Freie Universität Berlin, Humboldt-Universität zu Berlin, Department of Psychiatry and Psychotherapy, Berlin Institute of Health, Berlin, Germany

**Keywords:** homelessness, mental illness, precarious housing, therapeutic facility, quality of life, housing quality, housing satisfaction

## Abstract

The growing social problem of homelessness and precarious housing situations has negative effects on psychological outcomes and quality of life (QoL) for mentally ill people. Despite a large body of research on QoL among homeless mentally ill people, research on housing satisfaction as a specific QoL domain and important outcome variable for treatment interventions is scarce. The purpose of this cross-sectional study is to investigate housing satisfaction among psychiatric patients in various housing situations. Out of 1,251 patients that were treated in the targeted facilities during the admission period, 540 agreed to participate (43.2%). 123 participants were excluded from the analysis due to missing data, resulting in a sample of *N* = 417. Housing satisfaction data was assessed in a subjective screening and differences in satisfaction levels between housing status groups were analyzed. As hypothesized, more normative housing situations reported higher housing satisfaction. Homeless participants and those living in socio-therapeutic facilities were associated with more psychological and physical distress resulting from their housing situation than domiciled and flat-sharing participants. Problems of reducing homelessness and improving housing support are highlighted, as well as opportunities for improving support, particularly in therapeutic facilities.

## Introduction

The deinstitutionalization and reformation of the psychiatric system in Germany in the 1970s aimed at improving the living conditions of people with mental illness by creating residential care facilities (1, 2). As a result, an increasing number of psychiatric patients now live in community settings and housing facilities, and a great body of research has been conducted to evaluate those settings and conditions (1, 3). A potential unintended consequence of this is that the deinstitutionalization of mental health care is nowadays associated with a growing number of people with mental illness living in precarious living conditions, as its association with a greater social, cultural, political and work-related exclusion is discussed (2, 4).

Such marginalized living circumstances are negatively associated with psychological wellbeing (5–8). Quality of life (QoL) measures have increasingly been used in psychiatric research in recent years to understand how mentally ill people experience non-institutionalized settings (9–11). Several studies reported that higher QoL in persons with mental illness is more generally associated with normative housing conditions. This encompasses community-based residences and family homes that foster independence, stability and commonness, as opposed to state hospitals (12–14). Focusing mainly on clients in therapeutic psychiatric facilities, studies found that clients in assisted living accommodation reported lower levels of life satisfaction than the general population (15). Increasing QoL was found to be interrelated to type of housing: whereas low QoL could be linked to large institutional settings, higher measures were associated with a “normalization” of the housing situation in sheltered accommodation (13).

Additionally, some authors argue that the deinstitutionalization can be considered one of the causal factors for the growing social problem of homelessness, as patients were released from hospitals without the necessary support to live on their own (16–18). The marginalized group of homeless people experiences discrimination, social exclusion, segregation from support networks and a lack of insurance, income and social support (16, 17, 19–22). Furthermore, homelessness is associated with significantly more mental and physical health problems compared to the non-homeless population; and with poorer negative health-related prognoses, a reduced effectiveness of recovery, and higher mortality in people with mental disorders (18, 19, 23, 24).

Previous studies on QoL in regard to housing circumstances showed that homeless persons and psychiatric patients, but most strikingly homeless people with a mental disorder report a significantly lower QoL than the general population (5, 9, 17). Additionally, homeless mentally ill samples reported lower QoL in several life domains, including finances, friends and family, as compared to domiciled and community-based care patients (19, 25, 26).

Despite fundamental research on QoL and homelessness or therapeutic facilities, comparative data of a range of housing forms in mentally ill samples is lacking. An adequate differentiation between different types of housing and homelessness is frequently overlooked (e.g., sleeping at other people's places). For example, being at risk of homelessness could have comparable associations with mortality and prevalence of psychiatric disorders as homelessness itself (17, 27, 28). A distinction between various types of housing for people with mental illness, such as living in therapeutic facilities or in at-risk-of-homelessness situations could give insight into specific challenges and needs of different housing circumstances. Further, this could help target approaches for minimizing the stress of mentally ill people individuals.

Although results suggest that housing satisfaction (HS) as a subscale of QoL may be an important outcome in its own and an important predictor of further outcomes, most studies focus on general QoL assessments rather than domain-specific analyses (29). Housing itself, however, plays a central role in the treatment of mentally ill people and constitutes a stabilizing basis for everyday functioning, social integration, health, and participation (28, 30).

Therefore, this study examined HS measures among psychiatric in-patients in different housing settings. It was hypothesized that people in more precarious and less normative housing situations would report lower HS.

## Methods

### Study Design and Participants

The study is part of the “WOHIN” project, a cross-sectional survey designed to investigate the housing situation, psychiatric morbidity and service use among psychiatric in-patients and day-clinic patients treated in the catchment area of the Psychiatric University Hospital Charité at St. Hedwig Hospital over a 6-months period (15th March−15th September 2016) [see further details in (31, 32)]. The hospital offers in-patient treatment for 192 patients on three general psychiatric wards, four specialized wards, and 5 day-clinics. The study was approved by the local ethics committee of the Charité Berlin (Number: EA1/291/15).

A total number of 1,251 patients were admitted and contacted by trained interviewers soon upon admission. 540 (43.2%) were willing to participate. A monetary incentive (5€) was offered, interviews averaged 1 h. All participants gave written consent before participating.

From the original sample, 120 participants were excluded because important data for the analyses were missing. Three participants who answered <75% of the HS screening items were further excluded, leaving a sample size of *N* = 417. Missing values were analyzed and replaced by imputed mean scores.

### Instruments

#### Housing Status

In order to assign participants into groups regarding their housing status, their predominant housing situation 30 days prior to admission was examined. Housing status was categorized into four groups: (1) domiciled patient group (*domiciled*, including rented apartment, residential property), (2) patient group in socio-therapeutic facilities (*facilities*, including assisted housing, therapeutic shared apartment and special-care home), (3) patient group sleeping at friends' or acquaintances' places (*shared-flat*), and (4) homeless patient group (*homeless*, including literally homeless, emergency shelter, homeless shelter, refugee shelter, and women's shelter).

#### Socio-Demographic Variables

A structured interview conducted by trained interviewers collected information on socio-demographic variables of gender, age, marital status, parenthood as well as education and employment. Mental disorder diagnoses were based on existing discharge records and provided by psychiatric clinicians on the basis of ICD-10 criteria (33).

#### Housing Satisfaction Screening

A subjective screening was conducted to acquire measures of HS. The compilation of the screening items was based on the expertise of different professional groups, including doctors, social workers, and caregivers. In a short test phase, patients evaluated the items in regard to comprehensibility. The screening consisted of 25 items addressing several aspects of HS, including contacts, satisfaction with certain housing aspects such as security or accessibility and subjective assessments on the interplay of housing and psychological and physical wellbeing. Respondents were asked to rate the items in terms of accordance with the presented statement on a four-point Likert-scale ranging from “doesn't apply at all” to “completely applicable.”

### Statistical Analysis

All statistical analyses were conducted using IBM SPSS Version 23.0 (34). Standard descriptive analyses of socio-demographic variables were calculated depending on the data scales. The Chi-square test of independence was performed to determine if there is a statistically significant relationship between housing status and suitable socio-demographic variables. Fisher's exact test was used when sample sizes were small and the observed value in the contingency table was less than five, ANOVA was performed in case of metric variables.

In order to assess HS, an exploratory principal component analysis (PCA) was conducted to extract the most important factors of the subjective screening. Subsequently, a Promax rotation was run because correlations between the dimensions were assumed. Ten items had to be excluded because they were undirected and couldn't be linked to an either negative or positive value judgment. A total of 15 items were examined, and data from all participants who completed the screening were included (*N* = 417). Reliability analyses were conducted using Cronbach's alpha to assess the internal consistency of each factor. A sum score of the relevant items was calculated for each participant as the mathematical expression of housing satisfaction.

To examine the impact of housing status on HS, a one-way analysis of variance (ANOVA) was conducted on the overall sum scores as well as on each factor alone. Shapiro–Wilk test was used to check the data distribution, and Levene's test examined homogeneity of variance. Welch's ANOVA was performed and interpreted when the classical ANOVA assumption of homogeneity of variance was violated. Games–Howell *post-hoc* analysis was run to gain differentiated information on group differences in HS.

## Results

### Housing Status

At the time of data collection, 62.4% of the participants were *domiciled* (*N* = 260), 16.3% lived in socio-therapeutic *facilities* (*N* = 68), 10.3% lived in *shared flats* (*N* = 43), and 11.0% were *homeless* (*N* = 46).

### Socio-Demographic Background

The average age was 42.11 years, ranging from 18 to 89. The sample included 178 (42.7%) women and 238 (57.1%) men, one person identified themselves as *other* (0.2%). 27.9% were married or in a relationship, and 36.9% reported to have at least one child. The majority of participants had a school diploma (86.7%). More than one third had no professional education (37.0%) and 77.8% were unemployed.

The participants differed significantly regarding age, school, and professional education and employment status.

As Table 1 shows, *domiciled* patients were likely to be male, older, in a relationship, to have at least one child, to have graduated from school as well as from a professional education, to be employed and to have a mood disorder. The *shared-flat* group did not show relevant differences from *domiciled* patients in terms of gender, family status, school education, and employment status as well as mood disorder diagnosis, but showed less professional education and included younger participants with fewer children. *Homeless* and *facilities* groups were more often male, single, and had no school diploma and professional education. Though unemployment was very high in all groups, *facilities* and *homeless* comprised the highest percentages with around 95%. They also showed higher rates of substance dependence, while diagnoses of psychosis, substance abuse, and intellectual disabilities was relatively high in all groups except *domiciled*.

**Table 1:** Group distributions and corresponding test results of socio-demographic variables.

**Variable** **(*N* = 417)**	**Domiciled group** **(*N* = 260)**	**Facilities group** **(*N* = 68)**	**Shared-flat group** **(*N* = 43)**	**Homeless group** **(*N* = 46)**	**ANOVA, Chi-square and Cramer's *V*** **or Fisher's exact test**
Gender: Female	119 (45.8%)	27 (39.7%)	20 (46.5%)	12 (26.1%)	*p* = 0.156
Age (in years)	44.95 (*SD* = 14.47)	41.09 (*SD* = 15.51)	31.56 (*SD* = 11.23)	37.43 (*SD* = 13.73)	*F*_(1, 415)_ = 38.91, *p* < 0.001
Family status^1^					*p* = 0.006
Married	36 (14.1%)	4 (6.0%)	7 (16.3%)	4 (9.1%)	
In a relationship	43 (16.9%)	5 (7.5%)	12 (27.9%)	3 (6.8%)	
Divorced/in separation	50 (19.6%)	8 (11.9%)	3 (7.0%)	10 (22.7%)	
Widowed	10 (3.9%)	4 (6.0%)	–	1 (2.3%)	
Single	116 (45.5%)	46 (68.7%)	21 (48.8%)	26 (59.1%)	
At least one child	106 (41.4%)	24 (35.8%)	9 (20.9%)	15 (32.6%)	$\chi_{\left(3\right)}^{2}$ = 7.26, *p* = 0.064 Cramer's *V* = 0.133, *p* = 0.064
School education^2^					*p* < 0.001
Advanced education (13 years of school)	105 (41.2%)	8 (12.7%)	15 (35.7%)	14 (31.3%)	
Intermediate diploma (10 years of school)	86 (33.7%)	25 (39.7%)	14 (33.3%)	9 (20.0%)	
Basic school qualification (9 years of school)	42 (16.5%)	13 (20.6%)	8 (19.0%)	8 (17.8%)	
Special needs school graduation	2 (0.8%)	2 (3.2%)	–	–	
No school diploma	20 (7.8%)	15 (23.8%)	5 (11.9%)	14 (31.3%)	
Professional education^3^					*p* < 0.001
University/college	55 (21.8%)	2 (3.0%)	6 (14.0%)	5 (11.1%)	
Apprenticeship	136 (54.0%)	26 (39.4%)	14 (32.6%)	12 (26.7%)	
None	61 (24.2%)	38 (57.8%)	23 (53.5%)	28 (62.2%)	
Employment status^1^					*p* < 0.001
Full-time	34 (13.4%)	1 (1.5%)	3 (7.0%)	2 (4.4%)	
Half-time	13 (5.1%)	1 (1.5%)	3 (7.0%)	–	
In training	4 (1.6%)	–	1 (2.3%)	–	
Pensioned	26 (10.2%)	2 (3.0%)	1 (2.3%)	–	
Unemployed	177 (69.7%)	63 (94.0%)	35 (81.4%)	43 (95.6%)	
Diagnoses					
Organic mental disorders	15 (5.8%)	5 (7.4%)	–	1 (2.2%)	
Psychosis	55 (21.2%)	22 (32.4%)	15 (34.9%)	17 (37.0%)	
Any substance dependence	96 (36.9%)	36 (52.9%)	14 (32.6%)	26 (56.5%)	
Any substance abuse	38 (14.6%)	14 (20.6%)	10 (23.3%)	9 (19.6%)	
Mood disorder	115 (44.2%)	8 (11.8%)	17 (39.5%)	10 (21.7%)	
Anxiety disorder	12 (4.6%)	2 (2.9%)	1 (2.3%)	1 (2.2%)	
Personality disorder	47 (18.1%)	17 (25.0%)	9 (20.9%)	8 (17.4%)	
Intellectual disabilities	2 (0.8%)	5 (7.4%)	2 (4.7%)	1 (2.2%)	

*Quantities and percentages are depicted for fulfilled variables (“yes”). N varies due to missing data. Cramer's V is only reported in case of significance*.

1 *N = 409*.

2 *N = 405*.

3 *N = 406*.

### Factors of Housing Satisfaction

A PCA was performed to identify the primary domains of HS. One item was ruled out due to insufficient correlations with other items, leaving 14 items for the analysis. Kaiser–Meyer–Olkin measure of sampling adequacy was 0.87, indicating a good factor analysis. Bartlett's test of sphericity represented sufficiently large correlations between items (*p* < 0.001). Factors were identified by eigenvalues over 1.0 and their explained variability above 10% (35, 36).

The Promax-rotated PCA revealed empirical justification for retaining a two-factor solution including 12 items. The first factor accounted for 43.7% of the variance in HS and was labeled *psychological and physical distress*. It included seven items regarding subjective estimations on the extent of housing situation impacts on physical and emotional wellbeing. The second factor, *housing comfort*, contributed 11.2% to the overall explained variability. Its five items describe satisfaction with certain aspects of the current housing. All item loadings were above 0.5 in their assigned factor. Table 2 shows the factor loadings of each item. Internal consistency was high for the first factor, Cronbach's alpha = 0.86, and acceptable for factor two, Cronbach's alpha = 0.79. Together, the factors accounted for 54.9% of the variance in HS.

**Table 2:** Factor loadings of the subjective screening items on examined housing satisfaction domains.

**Item**	**Factor 1:** **Psychological and** **physical distress**	**Factor 2:** **Housing** **comfort**
Due to my housing situation, I have experienced violence.	0.698	
My housing situation is my biggest problem at the moment.	0.790	
I consider my housing situation to be very burdensome.	0.775	
My housing situation worsened my mental condition.	0.839	
My housing situation worsened my physical condition.	0.842	
Due to my housing situation, I feel lonely and excluded.	0.611	
I am satisfied with the safety of my housing situation.		0.770
My housing situation offers sufficient opportunities for quiet and relaxation.		0.733
I am satisfied with the possibilities for body care at my housing.		0.665
I am content with the amount of privacy at my housing.		0.695
I am satisfied with the accessibility of my housing in regard to my work place, friends and family.		0.782
I feel restricted by the rules of my residential facility.	0.562	

*The items were translated from German into English for depiction purposes only. Items were named in order of appearance in the original subjective screening. Factor loadings are depicted above 0.20*.

### Housing Satisfaction

A one-way ANOVA was performed to assess the effects of housing status on HS. Data was not normally distributed (Shapiro–Wilk test, *p* < 0.05). However, the ANOVA is robust against the violation of that requirement in case of a sufficiently large sample size (*N* ≥ 30). Furthermore, data violated the assumption of homogeneity of variances (Levene's test, *p* = 0.001) and thus, Welch's ANOVA results were interpreted.

As can be seen in Table 3, the level of overall HS differed significantly for the different types of housing status (*p* < 0.001). Games–Howell *post-hoc* analysis revealed significant differences between HS of all four groups.

**Table 3:** Descriptive housing satisfaction, including mean scores and standard deviation, and Welch's ANOVA results.

**Housing satisfaction** **(*N* = 417)**	**Domiciled group** ***(N* = 260)**	**Facilities group** ***(N* = 68)**	**Shared-flat group** ***(N* = 43)**	**Homeless group** ***(N* = 46)**	**Welch's *F***
Overall (max = 48)	40.06 (7.16)	36.19 (8.51)	36.63 (10.07)	28.73 (7.61)	Welch's *F*_(3, 99.29)_ = 30.70, *p* < 0.001
Factor 1 (max = 28)	23.81 (4.75)	20.90 (5.66)	21.47 (6.29)	17.45 (5.86)	Welch's *F*_(3, 98.19)_ = 19.63, *p* < 0.001
Factor 2 (max = 20)	16.24 (3.36)	15.29 (3.94)	15.16 (4.32)	11.28 (4.34)	Welch's *F*_(3, 98.13)_ = 18.32, *p* < 0.001

Table 3 further shows the averaged HS scores for each group. Level of HS regarding overall scores decreased from *domiciled* to *shared-flat* (−3.43, 95%-CI: [−7.68, 0.82]), from *shared-flat* to *facilities* (−0.43, 95%-CI: [−5.29, 4.42]), and from *facilities* to *homeless* (−7.45, 95%-CI: [−11.44, −3.47]). Significant group differences in HS were found from *domiciled* to *facilities* (*p* = 0.005), from *domiciled* to *homeless* (*p* < 0.001), and from *facilities* to *homeless* (*p* < 0.001). The *shared-flat* group had a significantly higher HS than *homeless* (*p* < 0.001), but not than *domiciled* (*p* = 0.153) or *facilities* (*p* = 0.995).

When differentiating between the two factors, Table 3 illustrates that the same pattern could be found for *psychological and physical distress* (*p* < 0.001). Satisfaction hereby decreased significantly from *domiciled* to *facilities* (*p* = 0.001), from *domiciled* to *homeless* (*p* < 0.001) and *facilities* to *homeless* (*p* = 0.012), while *shared-flat* only scored significantly higher than *homeless* (*p* = 0.013). When regarding the second factor shown in Table 3, *housing comfort* showed diverging trends (*p* < 0.001). Significant declines were found from *domiciled* to *homeless* (*p* < 0.001), from *facilities* to *homeless* (*p* < 0.001), and *shared-flat* to *homeless* (*p* < 0.001). *Shared-flat* could not be differentiated from *domiciled* (*p* = 0.403) nor from *facilities* (*p* = 0.999). Additionally, there were no differences between *domiciled* and *facilities* (*p* = 0.264), implying that only *homeless* differed significantly from all other groups in terms of *housing comfort*.

## Discussion

### Main Findings

To our knowledge, this is the first study to comprehensively evaluate HS across all housing situations of mentally ill people, rather than simply homeless vs. domiciled. As hypothesized, the results demonstrate a decrease in HS when the housing situation becomes less normative, which contributes to findings of other studies (12, 14, 19, 25). HS was found to be significantly higher for *domiciled* and *shared-flat* categories, than *facilities*, and finally, people in the *homeless* category reported the lowest satisfaction. These findings emphasize the heavy psychological burden of precarious living conditions and further illustrate the negative impact of homelessness on psychological wellbeing. Interestingly, participants living with friends or acquaintances show higher levels of overall HS, despite being considered as precariously housed. This form of housing is less independent and secure, due to a greater potential for conflicts, a need for compromise as well as a greater risk of having to move out in the case of temporary or uncertain leasing agreements. However, the familiarity of living in a household with others and the potentially supportive interactions with flatmates might protect the individuals from feeling psychologically or physically burdened by their housing situation. Additionally, HS of people living in shared flats might be higher due to aspects of autonomy and choice, which is further discussed below. These findings suggest that precarious living circumstances require nuanced analyses and reinforces the need for a more thorough understanding of the impact of different housing forms as well as opportunities for improvement.

When considering the overall scores of HS in all four groups, it is apparent that despite significant variations, the relative differences between group scores do not appear to be exceptionally large (i.e., *domiciled*−83.5% vs. *shared-flat*-−76.3%). These score proximities might be a result of a similar level of vulnerability and instability in our sample stemming from common mental health risks. Greater social burdens in all four groups, exemplified by exceptionally high unemployment rates, could further explain the lower group differences, as all groups seem to experience social precariousness to some extent. Furthermore, the total HS scores illustrate that no housing group reported a particularly low HS. The *facilities* group, for instance, achieved 75.4%, and the *homeless* group scored 59.8% of the total score. As HS is a subjective experience, it might be influenced by the duration of the current housing situation and the adaptation to its accompanying circumstances. The participants might have experienced several housing interventions due to a long history of mental illness, which could lead to a greater acceptance of housing facilities in general. As continuity of care throughout various elements of the service systems has been positively associated with QoL and greater service satisfaction, it could possibly further have an influence on HS specifically (37). In addition, long-term homelessness might influence the perception of different shelters, such as homeless shelters compared to emergency shelters. Generally, these results can also be understood in terms of the satisfaction paradox, which describes findings of relatively high housing satisfaction despite a low estimation of objective housing quality (38). These authors posit that this inconsistency could derive from resignation and cognitive dissonance caused by a perceived lack of opportunity for change. Housing should therefore be considered as an important resource in therapeutic processes.

The overall group differences are particularly apparent in the first factor on *psychological and physical distress*. The potential for constructively dealing with mental and physical health issues, social exclusion, restrictions, and negative experiences is notably lower for homeless patients, probably because homelessness is often accompanied by a lack of beneficial support and connection to helpful resources, while basic needs and the anticipation of discrimination become more present and important (39). Participants in socio-therapeutic facilities, however, also show lower levels of satisfaction in the first factor specifically, although these facilities are meant to target those aspects and to support their clients with social and health-related issues (40).

These group differences in HS might be explained by research on housing for mentally ill people, which proposes that characteristics of independence, choice, and control are important for psychopathology and QoL (23, 41–43). Further studies suggest that independent living in one's own dwelling increases HS in formerly homeless samples as opposed to dependent housing or group homes (11, 44). While research does concentrate on supported housing for mentally ill people with experiences of homelessness, studies on the effectiveness of supported housing forms for non-homelessness samples are scarce (40). However, the S3 guideline for psychosocial therapies for severe mental disorders (40) suggests avoiding permanent and high levels of institutionalization for people with mental illness in general as it increases negative or unwanted side effects. The separation of housing subvention and psychiatric or social care is highlighted and independent housing promoted as a means of enabling individual support regardless of residence.

These characteristics could play a role in this study's findings of diverging HS, because homeless people experience limited choices due to poverty and inequity, while patients in socio-therapeutic facilities deal with less autonomy and imposed rules (45). On the contrary, qualitative assessments of housing preference concluded that living in supported housing was associated with privacy and control as well as feeling secure and safe (46, 47). Furthermore, when examining the socio-demographic background, these participant groups show higher rates of psychosis and substance dependence and were less likely to be in a partnership or married. A difficult housing situation may be more salient in the case of people with greater psychological symptomatology or a scarce sense of wellbeing. Additionally, psychotic symptoms and substance procurement present competing priorities, which may overshadow concerns with one's housing (39). Conversely, previous negative housing experiences could amplify present symptomatology and substance use.

As several studies point out, there is a strong preference among persons with mental illness for living in their own apartment as it implies more independence and autonomy (43, 48). However, whether these aspects moderated the perception of psychological and physical stresses in this sample could not be analyzed in the current study. The relationship between those stresses and the suggested characteristics of independence, choice, and control as well as their role in socio-therapeutic facilities need to be investigated further. Potential moderating effects of the proposed characteristics could be integrated into HS measures to further explore the interrelation of the different factors.

The second subscale, *housing comfort*, represents housing features that ensure basic qualitative requirements of housing. Only the *homeless* group reported significantly lower satisfaction, whereas the groups *domiciled, facilities*, and *shared-flat* did not show any group differences. Since homeless people do not experience proper housing in any form, basic needs for safety, relaxation, hygiene, privacy, and accessibility in housing can naturally not be satisfied.

These findings support approaches such as Housing First to reduce homelessness and increase stable housing conditions (49). This intervention prioritizes the immediate and unconditional access to appropriate housing before further therapeutic support and focuses on recovery-oriented and support-oriented individual needs to build a basis for increased choice and decision freedom (49, 50).

### Limitations

The findings cannot be generalized to other regions because situational and local supply conditions can have an impact on housing opportunities, mental health risks, living circumstances, support networks, and health insurance for the targeted group (25, 31, 51). Secondly, the homeless sample is only specifically representative of those in psychiatric hospital care. While a high amount of homeless people suffer from mental illnesses, only a small number of them manage to seek professional help due to a high severity of symptoms and problematic living circumstances (18, 28). Affected individuals without psychiatric care might report diverging needs and challenges in everyday life. Additionally, housing forms of eligible patients who did not participate in the study could not be assessed due to refusal to participate, short lengths of stay or inability to consent. Potential associations between participation and housing form might impact our results and their representativeness and interpretation. Schreiter et al. (31) discuss differences between participants and non-participants in terms of age, gender, and certain diagnoses in more detail. Further, while acceptable reliability of the HS instrument was achieved and objectivity was ensured by standardized administration situations, validity of the developed scale has not been examined. To our knowledge, no validated German instrument on housing satisfaction would have been appropriate for all housing forms in our sample. The use of this individually tailored instrument limits psychometric testing and interpretation. A comparison to further HS research should be sensitive to possible impacts of differing measurements.

Given these findings on psychological and physical distress imposed by different housing situations, qualitative approaches on HS research might be appropriate for further exploration of this theme, providing more detailed insight into the reality of life of precariously housed persons.

## Conclusion

The insecurity and stress of homelessness and precarious housing contribute a serious additional burden to the lives of highly vulnerable mentally ill people. The findings highlight the importance of interventions targeting homelessness among psychiatric patients. The reported psychological and physical stress among people in therapeutic facilities underlines the need for adequate psychological support in such housing forms, to enhance HS among its clients.

## Data Availability Statement

The data analyzed in this study is subject to the following licenses/restrictions: data security reasons (identifiable data). Requests to access these datasets should be directed to Stefanie Schreiter (Stefanie.schreiter@charite.de).

## Ethics Statement

The study involving human participants were reviewed and approved by local ethics committee of the Charité—Universitätsmedizin Berlin. The patients/participants provided their written informed consent to participate in this study.

## Author Contributions

SS and SG were responsible for drafting and revising the original study protocol, they were the chief investigators and had overall responsibility for management of the trial, they delivered the training to the interviewers. MS-O revised the original study protocol and provided additional clinical supervision. EW wrote the framework and analysis plan. EW and JM analyzed the data. EW wrote the first draft of the report. SS, SG, and JM revised subsequent draft. All authors contributed to and approved the final report.

## Conflict of Interest

The authors declare that the research was conducted in the absence of any commercial or financial relationships that could be construed as a potential conflict of interest.
